# How to develop causal directed acyclic graphs for observational health research: a scoping review

**DOI:** 10.1080/17437199.2024.2402809

**Published:** 2024-09-27

**Authors:** Louise Poppe, Johan Steen, Wen Wei Loh, Geert Crombez, Fien De Block, Noortje Jacobs, Peter W. G. Tennant, Jelle Van Cauwenberg, Annick L. De Paepe

**Affiliations:** a Department of Public Health and Primary Care, Ghent University, Ghent, Belgium; b Department of Internal Medicine and Pediatrics, Ghent University, Ghent, Belgium; c Department of Intensive Care Medicine, Ghent University Hospital, Ghent, Belgium; d Renal Division, Ghent University Hospital, Ghent, Belgium; e Department of Methodology and Statistics, Maastricht University, Maastricht, The Netherlands; f Department of Quantitative Theory and Methods, Emory University, Atlanta, GA, USA; g Department of Experimental Clinical and Health Psychology, Ghent University, Ghent, Belgium; h Department of Movement and Sports Sciences, Ghent University, Ghent, Belgium; i Institute for Physical Activity and Nutrition, Deakin University, Melbourne, Australia; j Leeds Institute for Data Analytics, University of Leeds, Leeds, UK; k Alan Turing Institute, London, UK; l School of Public Health, Université Libre de Bruxelles, Brussels, Belgium

**Keywords:** Directed acyclic graph, causal diagram, causal inference, guidelines, recommendations, development

## Abstract

Causal directed acyclic graphs (DAGs) serve as intuitive tools to visually represent causal relationships between variables. While they find widespread use in guiding study design, data collection and statistical analysis, their adoption remains relatively rare in the domain of psychology. In this paper we describe the relevance of DAGs for health psychology, review guidelines for developing causal DAGs, and offer recommendations for their development. A scoping review searching for papers and resources describing guidelines for DAG development was conducted. Information extracted from the eligible papers and resources (*n* = 11) was categorised, and results were used to formulate recommendations. Most records focused on DAG development for data analysis, with similar steps outlined. However, we found notable variations on how to implement confounding variables (i.e., sequential inclusion versus exclusion). Also, how domain knowledge should be integrated in the development process was scarcely addressed. Only one paper described how to perform a literature search for DAG development. Key recommendations for causal DAG development are provided and discussed using an illustrative example.

## Background

Understanding causal relationships is a key aim of health research, in particular for theory building, and developing and evaluating interventions. Experiments and randomised controlled trials are considered the gold standard for interpreting associations between variables as cause–effect relations (i.e., for inferring causality) (Deaton & Cartwright, 2018). In many situations, such studies are not feasible or suitable because of ethical and practical challenges, or because interest lies in real-world settings (Rohrer, 2018). For that reason, researchers often rely on observational designs for inferring cause–effect relations (Rohrer, 2018).

Inferring causality from observational designs is notoriously challenging, because causal interpretations of associations are not guaranteed. Due to a lack of randomisation, an association between the independent variable (the exposure) and dependent variable (the outcome) might be the result of a common cause of both variables (i.e., *confounding bias*) (Glymour, 2006). For example, an association between meeting the WHO guidelines regarding moderate-to-vigorous physical activity (MVPA) (World Health Organization, 2020) and working memory (WM) performance in older adults does not necessarily reflect a causal effect of meeting the MVPA guidelines on WM performance. It could also be explained by age, a common cause of both variables. Consequently, the validity of causal interpretations relies on whether confounding has been sufficiently eliminated. Furthermore, interpretations of cause–effect relations may be hampered by the selection of participants on variables influenced by (common causes of) exposure and outcome characteristics. Expanding our example, suppose that participants were recruited via elderly care facilities, where residents typically have less physical and/or cognitive health. Consider a resident who meets the guidelines for MVPA, it is then more probable that this resident was admitted due to challenges in WM performance. Conversely, residents with a good WM performance, are more likely to have been admitted because of limited engagement with MVPA. Consequently, in this sample, one is likely to find a negative relation between the exposure (reaching the MVPA guidelines) and the outcome (WM performance). Such relation may be absent or even in the opposite direction in the general population of elderly. A distorted (possibly inversed) relation between exposure and outcome may thus occur because of endogenous *selection or collider bias*, a type of bias that, unlike confounding bias, is much less well-known among researchers in psychology (Glymour, 2006). In an experimental design, collider bias is often eliminated by sampling from a well-defined population before randomising participants. Besides confounding and collider bias, measurement bias (also called information bias) may arise in observational studies, and hamper causal interpretations of observational associations (Hernán, 2009). For example, reaching the MVPA guidelines may be measured with a questionnaire asking older adults to think back about the time they spent in several physical activities (e.g., walking, biking, etc.) over the past seven days (Craig et al., 2003). In this measure of MVPA, the true MVPA level will be distorted by other factors, including the individual’s ability to recall the MVPA undertaken over the past seven days. Measurement error (at least in the exposure) is generally prevented in randomised studies because participants are randomly assigned to well-defined exposure/treatment levels.

Awareness of these biases, especially confounding bias, has fostered a culture of caution regarding causal inference in observational research. As a result, many researchers have learned to avoid describing their aims and results in terms of ‘causal effects’, and write about (adjusted) ‘associations’ (Grosz et al., 2020; Hernán, 2018). Many researchers have also learned that adjusting for a large set of covariates is ‘a good practice’ to reduce confounding bias, often with the implicit belief that ‘more is better’ (Gaskell, 2020). However, adding covariates without considering the underlying causal structure may increase rather than reduce bias. In cases where causal effects are of interest, which often is the case, there is a need to employ more formal and systematic approaches that delineate the underlying causal assumptions and the associated analytical methods.

**Figure 1.**
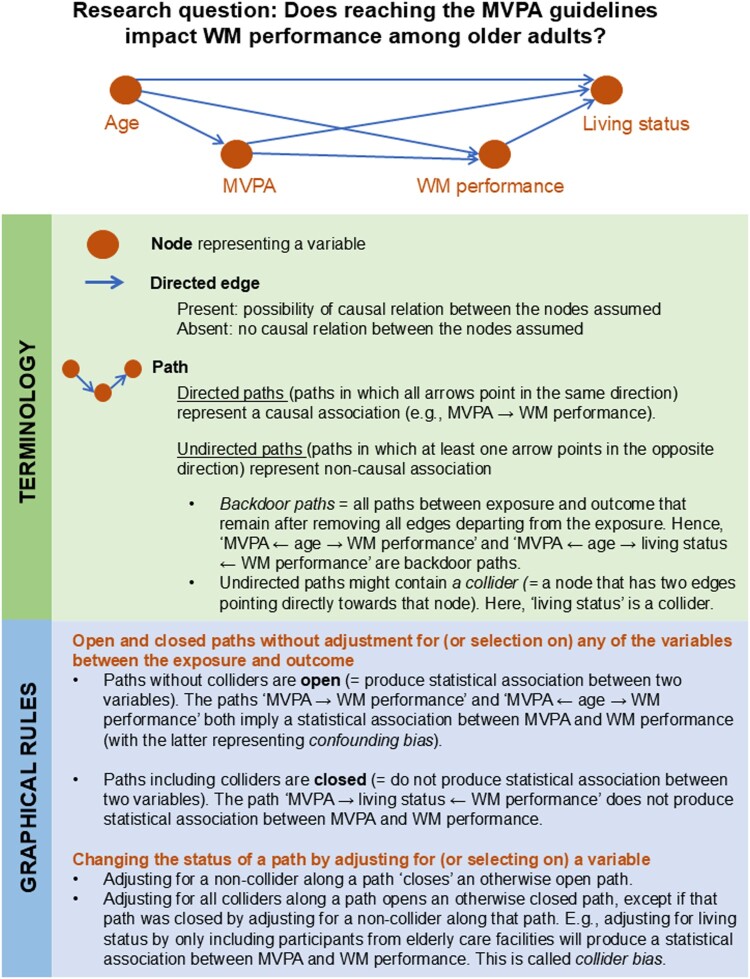
Basic definitions and graphical rules for Directed Acyclic Graphs. Notes. Measurement bias can be visualised using different structures. Interested readers are referred to Chapter 9 of Hernán and Robins (Hernán & Robins, 2020).

One such approach is the utilisation of causal Directed Acyclic Graphs (DAGs). These causal diagrams are invaluable tools for planning and conducting studies that aim to uncover causal effects in observational data (Greenland et al., 1999; Pearl, 2009). When our aim is to examine causal relationships, we need to go through two phases. In the identification phase, we conceptually define the causal structure between our variables based on background knowledge and decide whether the causal effect of interest can be identified from the available data under the assumed causal structure. In the estimation phase, we perform the statistical analysis using information derived from the identification phase. DAGs are highly relevant tools for the first phase as they allow us to provide an explicit and transparent visual representation of relevant background knowledge and the research design. A DAG can easily be scrutinised by fellow researchers, and provides a clear basis for discussion. Furthermore, DAGs allow a more formal approach to identify (DAG-implied) adjustment sets that reduce confounding bias and avoid inducing collider bias (due to over-adjustment or selective sampling of individuals from the target population). General introductions to causal DAGs are readily available; see e.g., Glymour, 2006; Hernán & Robins, 2020, Chapter 6; Morgan & Winship, 2015, Chapter 3; Elwert, 2013; Glymour et al., 2016). Figure 1 illustrates key definitions and graphical rules of DAGs, employing an example from health psychology. Although causal DAGs are increasingly used by epidemiologists and methodological specialists for guiding study design, data collection and adequate statistical analysis, they remain relatively rare in the health sciences (Tennant et al., 2021), and the behavioral and social sciences.

There may be several reasons for the slow uptake of causal DAGs in health psychology. First, explicit causal inference is often considered taboo in nonexperimental research in psychology (Grosz et al., 2020). Due to the prevailing mantra that only randomised experiments can uncover causal relationships, researchers conducting observational studies refrain from using causal language in reporting study aims and results. As a result, associational rather than causal analyses are conducted. Paradoxically, many researchers, even those using observational designs, are interested in answering causal questions, with causal implications inevitably surfacing when translating findings into policy and practice recommendations (Grosz et al., 2020; Hernán, 2018). Such (adjusted) ‘associational’ approaches are prone to the biases outlined above, and of limited value for health decision making (Crutzen & Peters, 2021; Glymour, 2006; Grosz et al., 2020; Hernán, 2018).

Second, structural equation models (SEMs) have a widespread use in psychology (Bollen & Pearl, 2013), and network analyses are currently experiencing an unprecedented surge in popularity (Borsboom et al., 2021). Both share (visual) similarities with causal DAGs (Bollen & Pearl, 2013; Borsboom et al., 2021; Huang et al., 2023). Hence, questions about the added value of the utilisation of causal DAGs arise. In observational studies, causal DAGs are primarily used as *input* to guide statistical estimation of a causal effect of a pre-defined exposure on a pre-defined outcome, and are, unlike network models, only rarely considered as the *output* of a statistical analysis. Unlike parametric SEMs and network models, causal DAGs are conceptual tools used primarily in the identification phase of causal analysis, and are not inherently linked to any specific statistical model or analytical technique. DAGs can hence be considered as non-parametric SEMs (Bollen & Pearl, 2013). In addition, SEMs are used for answering both causal and non-causal research questions (Kunicki et al., 2023). Network analysis is used to examine patterns of conditional (in)dependence between variables. Sometimes these patterns are used to develop hypotheses regarding the causal relations between variables or even to learn the underlying causal DAG from the available data (Ryan et al., 2022). However, this endeavour (sometimes termed ‘causal discovery’) is challenging (and arguably much more ambitious than causal effect estimation), not only because edges in network models can reflect non-causal correlations by adjusting for colliders, but also because it fundamentally relies on the strong assumption that all common causes of any pair of variables are included in the network model and hence captured in the available data (Ryan et al., 2022).

**Figure 2.**
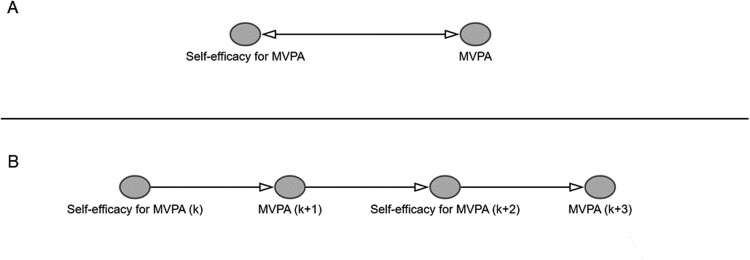
Panel A displays a feedback loop. Panel B shows the corresponding causal DAG encoding the temporal nature underlying the feedback loop. Notes. ‘k’ refers to timepoint k.

Third, because of their acyclic nature, researchers may believe that causal DAGs are not able to represent bidirectional effects between exposures and outcomes over time and are therefore ill-suited for capturing numerous phenomena of interest to health psychologists. This idea may have been reinforced by the fact that DAGs are often introduced with over-simplified examples with time-fixed exposures and single outcome measurements. Nevertheless, by considering and encoding the temporal and longitudinal nature of variables, DAGs can encode bidirectional effects characterised by ‘feedback loops’ between exposures, outcomes, and confounders (see Figure 2 for an example) (Hernán & Robins, 2020; Murray & Kunicki, 2022). In doing so, DAGs provide insight into the complex nature of potential biasing pathways that may operate through feedback loops between exposures, outcomes and time-varying confounders (Hernán & Robins, 2020).

Fourth, although the mathematical underpinnings of DAGs are well documented, guidance for applied researchers aiming to develop a DAG for answering their causal research questions is limited. Lack of knowledge regarding DAG development is an often reported barrier among researchers for adopting causal DAGs in empirical research (Barnard-Mayers et al., 2021). Furthermore, this lack of information can result in the development of highly implausible DAGs or the failure to use the developed DAG to (properly) guide further data-analysis (i.e., the estimation phase) (Barnard-Mayers et al., 2021).

This paper aims to further enhance the use of DAGs in the domain of health psychology by summarising the guidelines and recommendations on causal DAG development in the literature via a scoping review. Based on the findings, we will discuss and formulate recommendations for the development of DAGs, and illustrate these using an example within the field of health psychology.

## Methods

This review is based on Arksey and O’Malley’s six-step framework for conducting a scoping review (Arksey & O'Malley, 2005) and the PRISMA extension for scoping reviews (Tricco et al., 2018). The completed PRISMA checklist can be found in Supplementary File 1. The protocol was uploaded to the Open Science Framework (OSF) on the 10th ofJune 2022 (osf.io/u42q9).

### Step 1 – Identifying the research question

For this review, we considered the question ‘which guidelines or recommendations are provided for the development of causal DAGs?’. The review therefore sought to identify and summarise papers and resources focused on offering guidelines on how to develop a DAG for a research question.

### Step 2 – Identifying relevant records

Relevant records (e.g., papers, book chapters, online courses) were identified in two ways. First, potential records were identified by searching bibliographic databases. For the concept ‘DAG’ we adopted the search strategy used by Tennant and colleagues in their review on the use of DAGs in applied health research (Tennant et al., 2021). The search strategy was adapted for three bibliographic databases: (1) Medline (PubMed), (2) EMBASE and (3) Web of Science (see Supplementary File 2). Only English-language papers and resources were considered. No restrictions were set for the research area, time since publication, or type of paper or resource. Papers and resources that (1) did not provide guidance on how to create a DAG or (2) focused on causal discovery were excluded. The most recent search was performed on the 11th of March 2022. Additional papers and resources were identified by hand searching the reference lists of the papers identified by the bibliographic searches.

### Step 3 – Selecting relevant records

Records retrieved from the three databases were exported to Rayyan software (Ouzzani et al., 2016). After removing duplicates, relevant records were identified in two phases. First, titles and abstracts were screened in duplicate by LP, ADP, and JVC. LP screened all the records while ADP and JVC each screened 50 percent. For each record, the researchers indicated whether the record should be included, excluded, or in case of doubt, the label ‘maybe’ was allocated. After this, the results were compared and disagreements were discussed. The full texts of the remaining records were then assessed for final eligibility by LP who also searched the reference lists for additional relevant papers and resources.

### Step 4 – Charting the data

In step 4, LP extracted the key information from each of the included papers and resources (Arksey & O'Malley, 2005). Regarding general information, this included publication year, author names, title, country of first author’s affiliation, and type of publication (e.g., article). Regarding the provided guidance and recommendations, information was extracted according to four themes: (1) the main purpose of DAG development (i.e., whether the identified paper or resource focuses on developing a DAG to guide study design, data analysis, or both), (2) proposed guidelines for DAG development, (3) guidance on the consultation of experts for DAG development, and (4) guidance on how to perform a literature search for DAG development. Because the included records do not report on empirical studies, no quality appraisal of the individual records was performed.

### Step 5 – Collating, summarising and reporting the results

In step 5, a narrative summary of the findings was developed focusing on three themes of particular relevance to applied health researchers. First, we describe the purpose of developing a DAG. Second, we discuss the similarities and differences between the papers and resources regarding the proposed steps for DAG development. Third, we summarise the guidance on how to obtain domain knowledge (i.e., background knowledge regarding how other variables are related to the exposure and outcome of interest) for the development of a DAG.

### Step 6 – Consultation exercise

In the final step, the aim and content of the review were discussed with applied health researchers, to ensure it best meets the needs of potential users. To achieve this, three members of the research team (LP, ADP, and JVC) organised workshops at the 19th annual meeting of the International Society of Behavioral Nutrition and Physical Activity (Phoenix, USA) and at Ghent University (Ghent, Belgium). During these workshops, the aim and the content of the review were presented and discussed. Participants were asked: (1) ‘What are facilitators for reading the scoping review?’ (2) ‘What are barriers for reading the scoping review?’ and (3) ‘Are there other relevant themes that we should discuss in the scoping review?’. Supplementary File 3 provides an overview of the characteristics of the attendees and of the feedback obtained and how we implemented this feedback in the final manuscript.

## Results

### Sample description

**Figure 3.**
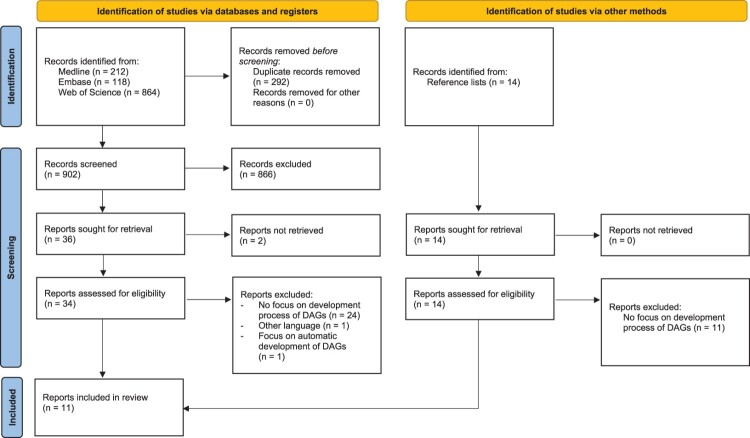
Completed PRISMA flow diagram.

Figure 3 shows the completed PRISMA flow diagram, which summarises the review selection process. A total of 902 unique records were identified from the three bibliographic databases. Of these, eight were included in the review. Fourteen additional records were identified from reference lists, of which three were included in the review. An overview of the eleven included papers and resources is provided in Table 1.

**Table 1.** Included papers and resources.

Authors (Date)	Country	Publication type	Title	Name of journal / book
Sauer and VanderWeele (2013)*	USA	Book supplement	Use of directed acyclic graphs	Developing a Protocol for Observational Comparative Effectiveness Research: A User’s Guide
Tafti and Shmueli (2020)	USA	Article	Beyond Overall Treatment Effects: Leveraging Covariates in Randomized Experiments Guided by Causal Structure	Information Systems Research
Grace and Irvine (2020)	USA	Article	Scientist’s guide to developing explanatory statistical models using causal analysis principles	Ecology
Gaskell (2020)	USA	Article	An Introduction to Causal Diagrams for Anesthesiology Research	Anesthesiology
Ferguson et al. (2020)	UK	Article	Evidence synthesis for constructing directed acyclic graphs (ESC-DAGs): a novel and systematic method for building directed acyclic graphs	International Journal of Epidemiology
Suzuki et al. (2020)*	Japan	Article	Causal diagrams: pitfalls and tips	Journal of Epidemiology
Tennant et al. (2021)	UK	Article	Use of directed acyclic graphs (DAGs) to identify confounders in applied health research: review and recommendations	International Journal of Epidemiology
Watkins (2021); Watkins (2022)	Australia	Conference abstract and website	An online searchable database of example causal diagrams to make them easier to construct	International Journal of Epidemiology
Laubach et al. (2021)	USA	Article	A biologist’s guide to model selection and causal inference	Proceedings of the Royal Society B
Digitale et al. (2022)	USA	Article	Tutorial on directed acyclic graphs	Journal of Clinical Epidemiology
Hernán (2022)*	USA	Online course	Causal diagrams: draw your assumptions before your conclusions	*NA*

Note: * = record identified via reference lists of the identified papers.

### Narrative summary

In the following paragraphs, we discuss (1) whether the included papers or resources focused on DAG development for guiding study design and/or data analysis, (2) the guidelines and recommendations proposed for DAG development, and (3) guidance provided for obtaining domain knowledge. Supplementary file 4 presents a summary of these themes for each of the included records.

#### Purpose of DAG development

Of the eleven included papers and resources, six focus on the development and use of DAGs for guiding data analysis (Ferguson et al., 2020; Grace & Irvine, 2020; Laubach et al., 2021; Sauer & VanderWeele, 2013; Tennant et al., 2021; Hernán, 2022). DAGs are indeed useful resources for gaining insight in the causal relations between variables and allow for the identification of sufficient adjustment sets, i.e., a set of variables that is sufficient for confounding adjustment of the exposure-outcome relation (Greenland et al., 1999). However, in their review, Tennant and colleagues found that many studies using DAGs do not report the DAG-implied adjustment set(s) and some studies even undermine the DAG-implied adjustment set(s) by adding or removing covariates from the analysis (Tennant et al., 2021). The authors suggested that lack of confidence or fear of criticism might cause these practices and recommended clearly reporting both the DAG and the DAG-implied adjustment set(s) (Tennant et al., 2021).

Five records highlight that DAGs also play a vital role in early phases of the research process and in guiding study design (Watkins, 2022; Digitale et al., 2022; Gaskell, 2020; Suzuki et al., 2020; Tafti & Shmueli, 2020). For example, Digitale et al. indicated that DAGs are valuable tools for conceiving research questions and are ideally reported in research proposals (Digitale et al., 2022). Additionally, DAGs allow researchers to prioritise which variables to measure for answering the causal questions of interest (Gaskell, 2020; Tafti & Shmueli, 2020). Consequently, limited time and resources are not wasted by measuring variables that are irrelevant or should not be adjusted for (Tafti & Shmueli, 2020).

#### Proposed guidelines and recommendations

Seven papers and resources outline specific steps for DAG development (Watkins, 2022; Laubach et al., 2021; Digitale et al., 2022; Ferguson et al., 2020; Gaskell, 2020; Tafti & Shmueli, 2020; Hernán, 2022). Four provide more general recommendations (Grace & Irvine, 2020; Sauer & VanderWeele, 2013; Suzuki et al., 2020; Tennant et al., 2021). The papers and resources providing specific steps for DAG development all indicate that the first step of creating a DAG consists of adding the exposure and outcome of interest and precisely defining both (Watkins, 2022; Laubach et al., 2021; Digitale et al., 2022; Ferguson et al., 2020; Gaskell, 2020; Tafti & Shmueli, 2020; Hernán, 2022). In two papers the authors argue that potential mediators should be included in the next step (Gaskell, 2020; Tafti & Shmueli, 2020). However, unless researchers are interested in the specific pathways of the effect from the exposure on the outcome (direct or indirect paths), including potential mediators is not a necessary step when focusing on the total effect (Hernán, 2022).

In the second step, common causes of exposure and outcome are added to the DAG. In the third step, common causes of each pair of variables included in the DAG in steps 1 and 2 are added (Watkins, 2022 Laubach et al., 2021; Digitale et al., 2022; Ferguson et al., 2020; Gaskell, 2020; Tafti & Shmueli, 2020; Hernán, 2022). The second and third steps are considered the most important aspect of developing a causal DAG (Sauer & VanderWeele, 2013). Importantly, when using DAGs to guide data analysis, the common causes integrated in the DAG should not be limited to variables available in the dataset, but should also include unmeasured variables when applicable (Watkins, 2022; Digitale et al., 2022; Ferguson et al., 2020; Gaskell, 2020; Sauer & VanderWeele, 2013; Tafti & Shmueli, 2020; Tennant et al., 2021; Hernán, 2022). Furthermore, to allow easier interpretation of the DAG and to avoid mistakes, Tennant et al. recommend to visually arrange the nodes in such way that they reflect the passage of time from left-to-right or from top-to-bottom (Tennant et al., 2021).

Further integrating these common causes in a DAG requires careful consideration. We identified two distinct approaches for this step in the selected literature: an additive approach and a dismantling approach. Six of the included records propose an additive approach in which the complexity of the DAG is gradually increased (Watkins, 2022; Laubach et al., 2021; Digitale et al., 2022; Gaskell, 2020; Tafti & Shmueli, 2020; Hernán, 2022). In this approach, after steps 1-3, the need to add directed edges between each pair of variables in the DAG is considered. For example, one record recommends to add a directed edge wherever theory or logic suggests a potential direct effect between two variables (Tafti & Shmueli, 2020). However, as absent arrows represent stronger assumptions than included arrows, Tennant et al. argue that providing a rationale for including edges is unnecessary (Tennant et al., 2021). Instead, the authors recommend justifying omitted edges with theory and/or evidence (Tennant et al., 2021). In line with this reasoning, the dismantling approach starts with including all potential edges and then gradually reduces the complexity of the DAG by removing edges (Ferguson et al., 2020; Grace & Irvine, 2020). For example, after applying steps 1-3, Ferguson et al. propose to draw edges between each pair of variables in the DAG, resulting in a saturated DAG (i.e., a DAG in which all variables are connected with all other variables in the DAG) (Ferguson et al., 2020). This step can be performed without considering the directionality of the edges linking the variables (Ferguson et al., 2020). Then, each of the edges are sequentially assessed and a rationale is provided for altering the direction of the edges (e.g., based on temporal order) or for removing edges (Ferguson et al., 2020). Making decisions regarding the direction of an edge between two variables that might influence each other in a DAG reflecting a cross-sectional design can be difficult. In this case, one resource advises that an edge should be drawn in the direction of the strongest causal effect (Watkins, 2022). Another option could be to develop multiple DAGs and to conduct sensitivity analyses testing the robustness of the results under different assumptions (i.e., by altering the direction of edges between the variables that might influence each other bidirectionally) (Ferguson et al., 2020; Sauer & VanderWeele, 2013).

In many realistic research settings, our analytical sample is not randomly selected from our target population. Selection bias is defined as ‘any bias away from the true causal effect in the referent population (i.e., the population before the selection process) due to selecting the sample from the referent population’ (Lu et al., [2022], page 699). Three records explicitly describe the inclusion of selection nodes in the DAG (Digitale et al., 2022; Gaskell, 2020; Hernán, 2022). By adding a selection node and drawing a box around this node researchers indicate that they implicitly stratify on this variable by focusing on a specific subsample of the referent population.

Measurement bias occurs when the relation between the outcome and the exposure is altered due to the process by which the data are measured (Hernán & Robins, 2020). Four records described the utility of DAGs to identify measurement bias (Digitale et al., 2022; Suzuki et al., 2020; Watkins, 2021; Hernán, 2022), but only one provided guidelines on how to represent measurement bias in DAGs. Adding nodes for mismeasured variables and edges representing causal influences on this mismeasurement is a final step in the DAG development approach proposed by Hernán (Hernán, 2022).

#### Guidance on how to obtain domain knowledge for DAG development

Decisions regarding which variables are relevant to include in a DAG should be based on domain knowledge (Ferguson et al., 2020; Hernán et al., 2002). As argued by Pearce and Lawlor, DAGs can only be as good as the domain knowledge used to develop them (Pearce & Lawlor, 2016). However, none of the included records describe the process via which domain experts are to be involved or consulted for DAG development. Similarly, guidance on how to perform a literature search for DAG development is scarce. On his website ‘causaldiagrams.org’ Watkins provides a searchable database of published health research literature including a causal diagram (Watkins, 2022). This database allows researchers to inform their DAG by previously created DAGs that focus on similar research questions. However, a DAG developed for one specific causal question in a specific context and for a specific population may not, without any modification, be copied to answer a similar causal question in a different context and population. Only one paper provided explicit guidance on how to perform a literature search for DAG development and how to incorporate the findings in a DAG. Ferguson and colleagues recommend starting the development of a DAG with a (novel) systematic review or a review of systematic reviews focusing on the ‘focal relationship(s)’, i.e., exposure-outcome relationship(s), of interest (Ferguson et al., 2020). Each of the studies identified via the systematic review or review of systematic reviews then goes through three stages. In the first stage, an implied graph is created. This implied graph is a saturated graph containing the outcome, the exposure and all ‘control variables’ for a specific study. The authors define control variables as variables that were included in the analysis assessing the relationship between the exposure and the outcome. In the second stage, each edge in the implied graph is evaluated based on causal theory. Hence, in this stage, edges can be reversed or removed. The final stage consists of integrating the directed edges and nodes from each of the implied DAGs into one DAG (Ferguson et al., 2020). Variables that were not identified in the retrieved literature, but are considered important by the researcher are also added in this step. The authors indicate that the final DAG might become very complex. Hence, researchers are advised to ‘recombine’ similar nodes. Similarity could be based on theory (e.g., the nodes are categories of one concept or are used interchangeably in the literature) or on conceptually related nodes having similar edges to and from other nodes (Ferguson et al., 2020). However, as the authors indicate, this approach is labour-intensive (Ferguson et al., 2020) and problems may arise when there is limited literature available for the specific research question.

## Discussion

Causal DAGs are tools to visually represent assumed causal relationships between variables. Importantly, they aid to make the causal assumptions invoked by researchers when causally interpreting their observational study results more transparent and explicit. Their uptake in psychology is limited, which might be due to a lack of knowledge and guidance on how to develop DAGs to inform study design and to guide data analysis. We therefore conducted a scoping review, examining papers and resources that describe guidelines and recommendations for their development. Our review revealed several areas of consensus, some less common suggestions, and some areas of disagreement.

Several resources highlighted the benefits of creating DAGs early in the research process, and not only for guiding data-analysis. When constructing DAGs, many recommended to include all common causes of both the exposure and outcome, regardless of whether they have been measured. There were varying recommendations on the optimal approach for including edges in DAGs. Some resources suggested an additive approach (e.g., Tafti & Shmueli [2020]), whereas others a dismantling approach (e.g., Ferguson et al. [2020]). Only a few resources recommended to incorporate nodes to represent participant selection procedures (Digitale et al., 2022; Gaskell, 2020; Hernán, 2022). Just one record offered guidance on adding nodes to represent measurement error (Hernán, 2022). Although many resources mentioned the importance of relying on expert knowledge in the field, there was very limited guidance on soliciting and integrating such domain knowledge during the DAG development process (see Ferguson et al. [2020] for a notable exception).

Based on these findings, we propose six steps when constructing a causal DAG (see Table 2). Given that constructing a DAG demands considerable time and investment, it is essential to allocate sufficient time to this process. Steps 1–5 comprise key recommendations distilled from our review. These steps focus on DAG development and hence refer to the identification phase of causal inference. In cases where the identified literature did not provide detailed guidance, we refer to the broad literature of causal inference. Step 6 provides guidance on how to use DAGs to inform data analysis, and is thus part of the estimation phase of causal inference. This step was added to ensure that the information derived from the DAG is actually used to analyse the data. To make the six-step procedure more tangible, a running example in the domain of health psychology is used. For each of the accompanying DAGs shown below, we provide a link to Dagitty, a free online tool not only for drawing, but also for analysing DAGs (Textor et al., 2011). Using this tool, interested readers can further explore how altering the DAGs changes the set of variables that should ideally be adjusted for in the estimation phase (see step 6).

**Table 2.** Six steps to consider when creating a DAG.

Step	Content
1	Create the DAG as early as possible in the research process
2	Precisely define the exposure and the outcome
3	Add common causes
4	Consider adding nodes representing selection procedures
5	Consider adding nodes representing measurement error
6	Use the DAG to inform data-analysis

### Step 1 – Create the DAG as early as possible in the research process

We recommend to construct a DAG as early as possible, preferably before the design of a study, the latest before data-analysis. Developing a DAG before designing the study permits researchers to select the variables that need to be measured and adjusted for (Greenland et al., 1999). Consequently, time and resources are not wasted by assessing variables that do not require adjustment (Tafti & Shmueli, 2020). Early DAG development may also help in refining selection procedures and measurement instruments to avoid potential sources of bias (e.g., by targeted recruitment of hard-to-reach subgroups). In case the data is already collected, it is also relevant to create a DAG in an early phase. Doing so will give relevant information on the need to link existing datasets from different studies to cover all relevant confounding pathways (see step 3) or to search for additional information from other sources to deal with selection (see step 4) or measurement (see step 5) bias.

*Illustrative example for step 1.* For exposition, we return to our example from the introduction section in which we examine the causal effect of reaching the WHO guidelines regarding MVPA on WM performance in older adults (World Health Organization, 2020). Here, we assume that we develop the DAG before conducting a large prospective longitudinal study among adults aged 65–80.

### Step 2 – Precisely define the exposure and the outcome

The first nodes to be added are the exposure and the outcome, which should be sufficiently well-defined and, ideally, separated in time. Longitudinal designs provide a means to temporally separate the exposure and outcome, allowing us to reduce the risk of reverse causality (i.e., the outcome causally affects the exposure). Of importance, a DAG focuses on the effect of one exposure on an outcome, i.e., the key research question at hand. If interest lies in the distinct effects of different (types of) exposures, separate DAGs should be constructed. For example, if we are also interested in whether alcohol consumption impacts WM performance, we should construct another, separate DAG. Furthermore, we should decide whether we are interested in examining the causal effect of an exposure at a single time point or an exposure (pattern) that accumulates over time. For example, we could examine the impact of reaching the MVPA guidelines at one timepoint (single time point exposure) or at two consecutive timepoints (cumulative exposure) on WM performance. Examining the impact of a cumulative exposure will make the DAG more complex as it requires adding multiple nodes to represent repeated measures of the exposure (and outcome) over time.

**Figure 4.**
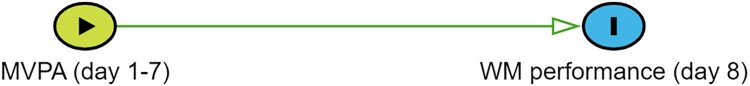
DAG depicting the exposure and outcome. Notes. https://dagitty.net/mgFapKirJ The green colour of the arrow indicates this edge captures an open directed path.

*Illustrative example for step 2.* We focus on the impact of reaching the WHO guidelines for MVPA (i.e., ≥ 150 minutes of MVPA per week [World Health Organization, 2020]) assessed over a period of one week on WM performance on the day following the assessment of our exposure. Note that MVPA will be assessed over a period of seven days, but that these data will be combined into a single binary variable (i.e., reaching or not reaching the WHO guidelines regarding MVPA). Hence, we have a single time point exposure and a temporal distinction between our exposure and outcome. The exposure and outcome are added to the DAG, and a directed edge is drawn from the exposure to the outcome (see Figure 4). Considering that the relationship between reaching the MVPA guidelines and WM performance could be bidirectional, one might also be interested in the effect of WM performance on reaching the MVPA guidelines. This would require the construction of a separate DAG for this specific research question and ideally measurements for WM would then precede rather than succeed those of MVPA. Both the exposure and the outcome will need to be further specified. Hence, we will have to decide how exactly we define MVPA (e.g., using metabolic equivalent of task) and WM performance (e.g., visuospatial or auditory WM performance).

### Step 3 – Add common causes

Adding common causes to the DAG requires domain knowledge obtained via literature and/or consultation of a domain expert. The evidence synthesis method proposed by Ferguson and colleagues allows researchers to gain an overview of variables that were considered relevant in studies by the original authors (Ferguson et al., 2020). However, scrutiny is required as variables in available studies might have been added to the analysis by the original authors without using a causal framework (and thus prone to introducing bias). The approach of Ferguson and colleagues also quickly becomes infeasible when substantive literature on a certain topic is available (Campbell et al., 2021). We recommend to start with a scoping review of articles with similar research questions, and list the common causes in these studies. A discussion with a group of experts may then follow about whether or not to include these in a DAG. To date, limited information is available regarding how to consult domain experts for DAG development. Possible methods are stakeholder workshops (Rodrigues et al., 2022) and publicly available feedback forms (Barnard-Mayers et al., 2022).

Researchers might wonder how many common causes their DAG should contain to be considered a ‘good’ DAG. There is no clear-cut answer to this question (Pearce & Lawlor, 2016). DAGs are always a simplification of reality. Their strength lies, however, in making this simplification explicit and open to scrutiny (Fleischer & Roux, 2008). Providing information on how domain knowledge was obtained (i.e., which domain experts and/or other resources were consulted) and incorporated will help reviewers and readers evaluate the quality of a given DAG, and prompt informed discussions. We recommend to document information on the development process of a DAG in detail in accompanying protocols and papers.

Edges linking the nodes can be added one by one (additive approach). Otherwise, a fully saturated DAG can be created, and implausible edges removed (dismantling approach). Organising the nodes in a way such that they represent the passage of time facilitates drawing the edges in the correct direction (Tennant et al., 2021). Irrespective of the approach used, it is essential to prioritise the justification of the omission of edges, rather than their inclusion. Furthermore, measured as well as unmeasured common causes should be considered (Morgan & Winship, 2015).

**Figure 5.**
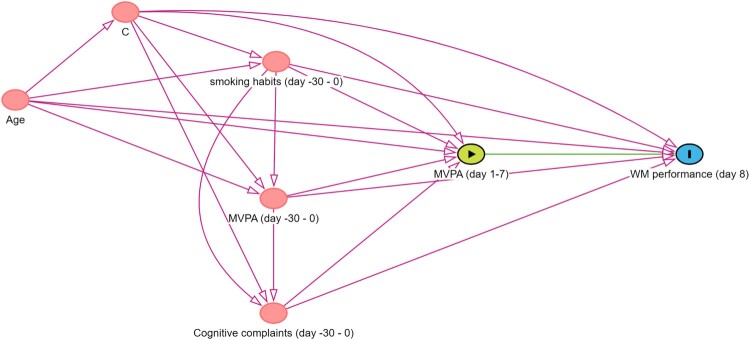
DAG with common causes. Notes. https://dagitty.net/mUqwRMept Arrows lying on an open undirected path are indicated in red.

*Illustrative example for step 3.* Common causes of MVPA and WM performance can be identified via a literature review and by consulting researchers from the domain of physical activity and/or cognitive functioning among older adults. A good example is provided by Campbell and colleagues, who used the evidence synthesis method of Ferguson et al. to construct a DAG outlining the relationship between physical activity and cognitive function (Campbell et al., 2021; Ferguson et al., 2020). Common causes included, amongst others, age and smoking habits (Campbell & Cullen, 2023). These variables are also likely to affect both reaching the MVPA guidelines and WM performance. Hence, age and smoking status are added to our DAG. As past MVPA and cognitive complaints are likely to influence the exposure and outcome, these variables are also added as common causes. Participants would be asked to consider the past month when reporting on their smoking habits, MVPA and cognitive complaints. For simplicity, the remaining common causes are represented by the node C. Figure 5 shows that adding the common causes creates multiple undirected or spurious (backdoor) paths between reaching the MVPA guidelines and WM performance.

Having a longitudinal design facilitates identifying a variable as a common cause or as a mediator. For example, sleep quality assessed before the assessment of MVPA can be considered a common cause, but not a mediator. However, sleep quality during the night following day 7 can act as a mediator, but not as a common cause. As we are interested in the total effect of reaching the MVPA guidelines on WM performance, mediators are not included in our DAG (Hernán, 2022).

### Step 4 – Consider adding nodes representing selection procedures

Selection bias often occurs as a result of stratifying on or adjusting for a collider, threatening the internal validity of the study. Selection nodes are not only useful to signal and visualise potential threats to interval validity, but also to external validity (Greenland, 2017; Hernán, 2017). Correcting for bias due to systematic selection of the analytical sample (from the target population of interest) usually requires information that is typically missing in the study data (e.g., to estimate the probability of selection in the study) (Hernán et al., 2004; Hernán & Robins, 2020; 2024). In contrast, when creating a DAG before designing the study, one might create more adequate designs to reduce the impact of such selection procedures; see (Ren & Loh, 2024) for examples from psychology research of how selection bias can arise.

**Figure 6.**
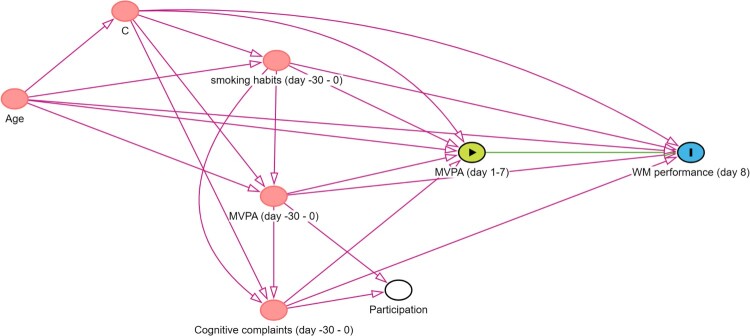
DAG including a collider. Notes. https://dagitty.net/mocqpRgwk

*Illustrative example for step 4.* When advertising a study examining the impact of reaching the WHO guidelines regarding MVPA on WM performance in older adults, one can imagine that people reaching these guidelines and people experiencing cognitive complaints are more likely to participate in the study. Figure 6 shows a DAG in which this selection procedure in visualised. The node ‘Participation’ indicates whether or not people participate in the study. We stratify on this variable as we will only have data from people who participated in the study. In doing so, we become aware of the undirected pathways between our exposure and outcome (i.e., MVPA (day1-7) ← MVPA (day -30–0) → Participation ← Cognitive complaints (day -30–0) → WM performance (day 8) and MVPA (day1-7) ← Cognitive complaints (day -30–0) → Participation ← MVPA (day -30–0) → WM performance (day 8)). This is also known as volunteer bias (Lu et al., 2022). Leaving this path open would threaten the internal validity of our study. Of interest, the spurious path can be closed by adjusting for ‘MVPA (day-30–0)’ or ‘Cognitive complaints (day -30–0)’. However, even though, in the absence of unmeasured common causes of participation and the outcome (and of participation and the exposure), this eliminates collider bias and hence removes the threat to internal validity, it does not eliminate the remaining selection bias (due to selective sampling) that may threaten external validity.

### Step 5 – Consider adding nodes representing measurement error

Hernán and Robins recommend first drawing a DAG assuming that all variables are perfectly measured. This allows researchers to focus on confounding and selection bias before considering the complexities related to measurement error. However, mismeasurement and its potential impact in the form of measurement bias should not be overlooked as measurement errors in the exposure, outcome, or important confounders can strongly bias the effect estimate (Hernán & Robins, 2020). By developing a DAG before designing the study, one can anticipate different sources of measurement bias and change the research design accordingly. Correcting for measurement bias after data collection requires (like selection bias) researchers to collect and model information that is usually not available in the original study data (e.g., from a validation (sub)sample that includes key variables measured without error) (Keogh, 2014; Keogh et al., 2020).

Psychologists are often interested in the causal effect of latent variables. Examining the causal effect of latent variables requires a precise definition of these variables and a clear justification for the methods used to assess these variables (VanderWeele, 2022). This is in line with recent calls for more conceptual clarification regarding measurement instruments and psychological constructs (Bringmann et al., 2022; Crombez et al., 2023; Crombez et al., 2024; Peters & Crutzen, 2024). The Decentralized Construct Taxonomy specifications outlined by Peters and Crutzen may be a helpful resource to transparently define constructs, and to provide instructions for their assessment (Peters & Crutzen, 2024).

**Figure 7.**
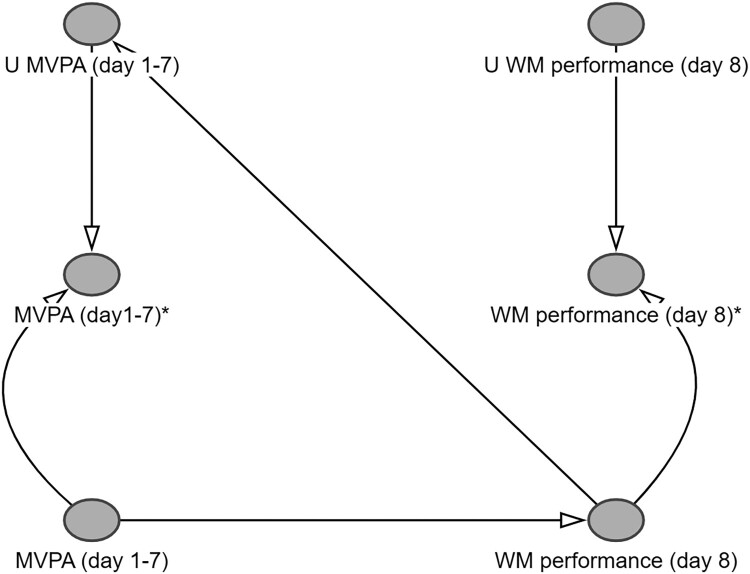
DAG representing measurement bias. Notes: https://dagitty.net/mWeagRxdW

*Illustrative example for step 5.* We could decide to assess older adults’ MVPA using a questionnaire asking participants to recall their physical activities in the past seven days (e.g., [Craig et al., 2003]) and their WM performance using neuropsychological tests. Figure 7 displays the relation between both variables. For simplicity, we will only focus on measurement error in the exposure and outcome. To avoid clutter, we only display these variables in the DAG. The nodes ‘MVPA’ and ‘WM performance’ refer to the perfectly measured variables, also known as latent constructs (Hernán & Robins, 2020). We will not have direct access to these values. Instead, we will have to use the measured values ‘MVPA*’ and ‘WM performance*’. These measures are influenced not only by the true latent variables but also by unknown measurement errors represented by ‘U_MVPA_’ and ‘U_WM_performance_’. Because the ability to correctly report physical activities over the past seven days may depend on WM performance, an arrow is drawn from the outcome to U_MVPA_. Consequently, MVPA* and WM performance* are expected to be associated even under the null hypothesis of no effect of the true exposure on the true outcome (Hernán, 2009). Hence, based on this DAG, researchers may decide to assess MVPA using a method that is less likely to be influenced by WM performance (e.g., accelerometry).

### Step 6 – Use the DAG to inform data analysis

In this step we move from the identification phase to the estimation phase. Different data-analytic methods can be used which can be broadly classified into three categories: regression adjustment (which focuses on modelling the relation between the outcome and exposure, conditional on confounders), propensity score methods (which primarily focus on modelling the relation between the exposure and confounders) and double robust methods (which focus on modelling both types of relations) (Hernán et al., 2019). Irrespective of the specific data-analytic method, the main aim is to close all spurious paths between the exposure and outcome while ensuring that no new spurious paths are opened. Hence, we aim to adjust for common causes, but not for colliders or mediators. This is a challenging job when we have a complex DAG. Fortunately, tools like Dagitty can help to automatically identify potential (or minimally sufficient) adjustment set(s) for estimating particular effects using regression adjustment, propensity score methods or double robust methods (Textor et al., 2011).

There are two important caveats related to the use of DAGs in the estimation phase. First, DAGs provide no guidance regarding the assumptions underlying parametric modelling (e.g., functional form of the relation between the variables in the DAG) (Digitale et al., 2022). Hence, well-considered statistical modelling decisions are still required in order to obtain consistent estimates of the exposure’s effect on the outcome. Second, because DAGs provide no information on the strength or the functional form of the relation between the included variables, the magnitude of different sources of bias is not captured by a DAG. Hence, DAGs may foster the detection and understanding of biases, but provide little information on the strength of these biases (Pearce & Lawlor, 2016). To overcome this limitation, researchers should conduct quantitative bias analyses, a form of sensitivity analyses which uses external data to provide more accurate effect and uncertainty estimates (Fox et al., 2022).

*Illustrative example for step 6.* In this step, we will assume that we were able to assess our variables without measurement error. The DAG displayed in Figure 6 indicates that we should adjust for Age, C, Cognitive complaints (day -30–0), MVPA (day -30–0), and smoking habits (day -30–0). An often used method is to include these variables as covariates in a linear regression model. This approach requires that each of the included variables has an additive and linear relationship with the outcome. Incorrectly modelled covariates will result in residual confounding.

Until now, we illustrated the six steps for the effect of a single time point exposure on a single time point outcome. As indicated in step 2, DAGs can be readily extended to represent multiple time point exposures (and outcomes). For example, in a longitudinal study, we could examine the impact of reaching the MVPA guidelines on two consecutive timepoints on WM performance. This type of research questions requires us to properly model time-varying confounders that may be affected by earlier exposures (also known as treatment-confounder feedback) and hence simultaneously act as mediators on certain causal pathways and as confounders on other causal pathways. For example, reaching the MVPA guidelines on week 1 might affect cognitive complaints at the end of that week, which in turn may affect reaching the MVPA guidelines on week 2. Hence, the time-varying confounder ‘cognitive complaints’ becomes both a mediator and a confounder. The issue of treatment-confounder feedback is also relevant to consider when deciding on whether a cumulative or sustained exposure (aggregated) over a longer time window (e.g., MVPA or diet assessed over one week) could be more adequately treated as a time-varying rather than a time-fixed exposure.^1^ Traditional regression adjustment and most propensity score methods (with the exception of inverse probability weighting) fail to properly account for treatment-confounder feedback and will generally produce biased effect estimates [Hernán and Robins (2020), Chapter 20]. G-methods, a family of advanced statistical methods (where the ‘g’ stands for generalised) developed by Robins and colleagues (Robins & Hernan, 2008), can be used to estimate the cumulative causal effects of exposure at consecutive timepoints (or more generally defined exposure regimes) (Hernán & Robins, 2020). Accessible introductions to g-methods have been introduced (Loh et al., 2024; Loh & Ren, 2023a); see Kennedy et al. for a recent application of one such method (Kennedy et al. 2023).

The six guiding steps outlined above illustrate that developing and using DAGs requires iterative discussions and considerable time investment. However, this brings some substantial benefits. First, it stimulates researchers to develop a better understanding of their research question and their underlying assumptions before collecting and/or analysing their data. Second, it fosters closer collaboration and cross-pollination by enhancing communication between researchers from different domains (Digitale et al., 2022). Consequently, developing DAGs to answer research questions fits within both the ‘Open Science’ movement, which focuses on transparency, rigour, reproducibility and replicability (UNESCO and Canadian Commission for UNESCO, 2022) and the ‘Slow Science’ movement, which invites researchers to think about the bigger aims of science (e.g., contributing to society) and advocates for less, but higher quality research (Altman, 1994; Frith, 2020).

### Limitations and future directions

There are some limitations to this review. First, although we sought to include all relevant resources, we may have missed papers and resources. However, including all papers and resources may not be necessary to provide a general overview of guidelines and recommendations. Second, the six steps outlined above cannot be considered an exhaustive checklist for DAG development. Nonetheless, we do believe that these six steps will provide researchers with a scaffolding, which will allow them to further delve into the growing DAG-literature.

Despite their advantages, DAGs also have limitations. First, DAGs do not replace the need to formulate a clear research question. In fact, predefining a precise target of causal inference (ideally a causal ‘estimand’ that is defined without making any reference to a particular statistical model) is indispensable for DAG development, for design-related and data analytical choices, and ultimately, for enabling unambiguous and well-balanced interpretations of study results (Lundberg & Johnson, 2021). Second, as indicated above, DAGs are acyclic by definition. Nonetheless, feedback loops can be represented by ‘unrolling’ the relation between variables over discrete timepoints (i.e., splitting single variable nodes into multiple sequential nodes to represent their longitudinal measurements) (Bongers, 2022). Health behaviors are, however, influenced by a continuous interplay between variables at different levels (intra-individual, inter-individual and contextual) and constantly changing and adapting over time and contexts (Chevance et al., 2021; Gomersall, 2018). In the past years there has been a plea to acknowledge the complex dynamics of health behaviors (Chevance et al., 2021; Gomersall, 2018; Heino et al., 2021). This shift is reflected in the rise of studies using intensive longitudinal designs (e.g., experience sampling methodology (Kuppens, 2021) capturing health behaviors over time and contexts. The application of DAGs for causal inference with intensive longitudinal data is, however, in its early stages. Furthermore, within the domain of psychology, mixed-effects models are often used to analyse longitudinal data. Currently, literature focusing on causal inference with longitudinal data analysed using mixed-effects models is scarce (although see Shardell and Ferrucci [2018] and Kim and Steiner [2021] for recent developments). The call to embrace the complex dynamics of health behaviors is also reflected in the adoption of modelling techniques that are able to deal with cyclic and nonlinear relationships between variables (e.g., formal, dynamical systems modelling (Perski et al., 2024). Recently, a framework of structural dynamical causal models (SDCMs) has been proposed to explicate the causal interpretation of dynamical systems (Bongers, 2022). Nonetheless, it is important to note that the fundamental causal diagrammatic issues raised in this paper, specifically the ability to nonparametrically identify causal effects of non-randomised exposures, apply generally in longitudinal designs with many time points. For example, even in the simplest possible scenario with just two time points, deciding whether to adjust or not to adjust for a previous outcome can be facilitated using causal diagrams (Loh & Ren, 2023b). Finally, DAGs are mainly used to examine causal relationships between well-defined and measurable variables (e.g., vaccination status). Psychologists are often interested in the effect of latent variables (e.g., self-efficacy for engaging in MVPA) on health behaviors. Further research on methods combining structural equation modelling, which acknowledges the presence of latent variables, and DAGs is expected to enhance causal inference within the domain of psychology (Mulder, 2024).

## Supplementary Material

Supplementary_File1.pdf

Supplementary_File2.pdf

Supplementary_File3.pdf

Supplementary_File4.docx

## Note

1 The longer the time window over which exposure levels are aggregated, the longer the window of opportunity for intermediate (non-aggregated) exposure levels to evolve and vary over time, and hence also for confounding factors to get entangled in treatment-confounder feedback loops, also within the considered time window. Acknowledging the original time-varying nature of time-aggregated exposures may therefore be warranted to adequately address (time-varying) confounding (and potential forms of time-dependent bias) within such longer time windows.

## Abbreviations

Abbreviations: DAG: directed acyclic graph; SEM: structural equation model; MVPA: moderate-to-vigorous intensity physical activity; WM: working memory; SDCM: structural dynamical causal model.
