# Supplementary material for: How to develop causal directed acyclic graphs for observational health research: a scoping review
Source: Health Psychol Rev. 2024 Sep 27;19(1):45–65. doi: 10.1080/17437199.2024.2402809 (PMC11875439; doi:10.1080/17437199.2024.2402809)
Supplement: Supplementary_File2.pdf [file RHPR_A_2402809_SM3522.pdf]

Supplementary File 2. Search strings for Medline (PubMed), EMBASE and Web of Science

Table 1. Search string for Medline (PubMed).

Concept	Search strategy
Concept 1: DAG1	"Directed acyclic graph"[tiab] OR "Directed acyclic graphs"[tiab] OR "causal diagram"[tiab] OR "causal diagrams"[tiab] OR "causal graph"[tiab] OR "causal graphs"[tiab] OR "graphical model theory"[tiab] OR DAGitty[tiab] OR "causal model"[tiab] OR "causal models"[tiab]
Concept 2: SEM	"structural equation modelling"[tiab] OR SEM[tiab] OR "path diagram"[tiab]
Concept 3: Building	building[ti] OR build[ti] OR built[ti] OR construct[ti] OR construction[ti] OR constructing[ti] OR recommend*[ti] OR develop[ti] OR development[ti] OR developing[ti] OR protocol[ti] OR create[ti] OR creation[ti] OR creating[ti] OR generate[ti] OR generation[ti] OR guide[ti] OR guiding[ti] OR guidelines[ti] OR guideline[ti] OR draw*[ti] OR design*[ti] OR practic*[ti] OR methodological[ti] OR introduction[ti] OR introducing[ti] OR demonstration[ti] OR demonstrating[ti] OR overview[ti] OR steps[ti] OR tutorial[ti]
Combination of concepts	(#1 NOT #2) AND #3

Note. tiab = keyword in title and abstract, ti = keyword in title

Table 2. Search string for EMBASE

Concept	Search strategy
Concept 1: DAG1	'Directed acyclic graph':ti,ab,kw OR 'Directed acyclic graphs':ti,ab,kw OR 'causal diagram':ti,ab,kw OR 'causal diagrams':ti,ab,kw OR 'causal graph':ti,ab,kw OR 'causal graphs':ti,ab,kw OR 'graphical model theory':ti,ab,kw OR DAGitty:ti,ab,kw OR 'causal model':ti,ab,kw OR 'causal models':ti,ab,kw

Concept 2: SEM	'structural equation modelling':ti,ab,kw OR SEM:ti,ab,kw OR 'path diagram':ti,ab,kw
Concept 3: building	building:ti OR build:ti OR built:ti OR construct:ti OR construction:ti OR constructing:ti OR recommend*:ti OR develop:ti OR development:ti OR developing:ti OR protocol:ti OR create:ti OR creation:ti OR creating:ti OR generate:ti OR generation:ti OR guide:ti OR guiding:ti OR guidelines:ti OR guideline:ti OR draw*:ti OR design*:ti OR practic*:ti OR methodological:ti OR introduction:ti OR introducing:ti OR demonstration:ti OR demonstrating:ti OR overview:ti OR steps:ti OR tutorial:ti
Combination of concepts	(#1 NOT #2) AND #3

Note. Ti = title, ab = abstract, kw = keyword

Table 3. Search string for Web of Science

**Concept**	**Search strategy**
Concept 1: DAG1	TS=("Directed acyclic graph" OR "Directed acyclic graphs" OR "causal diagram" OR "causal diagrams" OR "causal graph" OR "causal graphs" OR "graphical model theory" OR DAGitty OR "causal model" OR "causal models")
Concept 2: SEM	TS=("structural equation modelling" OR SEM OR "path diagram")
Concept 3: building	TI=(building OR build OR built OR construct OR construction OR constructing OR recommend* OR develop OR development OR developing OR protocol OR create OR creation OR creating OR generate OR generation OR guide OR guiding OR guidelines OR guideline OR draw* OR design* OR practic* OR methodological OR introduction OR introducing OR demonstration OR demonstrating OR overview OR steps OR tutorial)
Combination of concepts	(#1 NOT #2) AND #3

Note. TS = topic, TI = title
