# Supplementary material for: How to develop causal directed acyclic graphs for observational health research: a scoping review
Source: Health Psychol Rev. 2024 Sep 27;19(1):45–65. doi: 10.1080/17437199.2024.2402809 (PMC11875439; doi:10.1080/17437199.2024.2402809)
Supplement: Supplementary_File3.pdf [file RHPR_A_2402809_SM3521.pdf]

Supplementary File 3. Results of the consultation exercise

A total of 25 PhD students and 12 postdoctoral researchers participated in the workshops. All attendees performed research within the domains of Health Psychology, Public Health and/or Epidemiology. Research topics included sleep, physical activity, sedentary behavior, mental health, alcohol consumption, smoking cessation and diet. The attendees had no or limited experience with causal inference or DAGs.

Table 1. Overview of feedback from potential end-users and implementation of feedback in the manuscript

Feedback	Implementation of feedback
Facilitators for reading the paper	
• Explanation on the difference between association and causation	• In the Introduction section we indicate that association may be induced, increased, reduced or obscured between an exposure variable and an outcome variable by different types of biases besides the exposure causing the outcome.
• References to introductory papers about causal inference and DAGs	• In the Introduction section we refer to accessible introductions to causal inference and DAGs.
• Examples demonstrating the applicability of DAGs in health research	• In the Introduction section, the concept of causal DAGs is explained using an example from health psychology research (i.e., the effect of reaching the physical activity guidelines on processing speed among older adults).
• Providing a stepwise approach for DAG development	• In the Discussion section we describe a stepwise approach for developing DAGs based on the recommendations

	and guidelines provided by the identified literature.
• Keeping feasibility of DAG development in mind	• The feasibility of DAG development is discussed in the Results section and the Discussion section.
Barriers for reading the paper	
• Extensive use of jargon / too complex	• Although we needed to introduce some DAG-specific jargon, several efforts were made to keep a good balance between writing an introductory paper and providing sufficient information.
• Limited links with the domain of health research	• In the Introduction section jargon related to DAGs is explained using an example from the domain of health psychology research.
Other themes that are relevant to include in the paper	
• Linking the use of DAGs with the concept of ‘Slow Science’	• In the Discussion section we link the use of DAGs with the ‘Slow Science’ movement.
• Linking the use of DAGs with the concept of ‘Open Science’	• In the Discussion section we link the use of DAGs with the ‘Open Science’ movement.
