# Supplementary material for: How to develop causal directed acyclic graphs for observational health research: a scoping review
Source: Health Psychol Rev. 2024 Sep 27;19(1):45–65. doi: 10.1080/17437199.2024.2402809 (PMC11875439; doi:10.1080/17437199.2024.2402809)
Supplement: Supplementary_File4.docx [file RHPR_A_2402809_SM3520.docx]

Supplementary File 4. Summary of the selected papers and resources

Table 1. Summary of the selected papers and resources

| Record | Purpose of DAG development | Proposed guidelines for DAG development | Guidance on consultation of experts? | Guidance regarding literature search? |
| --- | --- | --- | --- | --- |
| Sauer & Vanderweele (2013) [1] | Focus on data-analysis | “*The first step in creating a causal DAG is to diagram the investigators’ understanding of the relationships and dependencies among variables. Construction of DAGs should not be limited to measured variables from available data; they must be constructed independent of available data and of background knowledge of the causal network linking treatment to the outcome. The most important aspect of constructing a causal DAG is to include on the DAG any common cause of any other two variables on the DAG. Variables that only causally influence one other variable (exogenous variables) may be included or omitted from the DAG, but common causes must be included for the DAG to be considered causal.*”; “*Investigators may not agree on a single DAG to represent a complex clinical question; when this occurs, multiple DAGs may be constructed and statistical associations observed from available data may be used to evaluate the consistency of observed probability distributions with the proposed DAGs. Statistical analyses may be undertaken as informed by different DAGs, and the results can be compared.*” | No | No |
| Tafti & Schmueli (2020) [2] | Focus on study design and data-analysis | *"1. Set the treatment node and outcome node(s). 2. Add nodes for measured pre-treatment variables. 3. Add nodes for potential mediating variables. These are variables of interest which, based on theory, lie on the causal path (i.e. directed path) from the treatment to the outcome. 4. Add nodes for unmeasured (i.e. unobserved) variables that may lie on the spurious path from any of the nodes to the outcome. 5. Add arrows to and from each variable wherever theory or logic suggests the possibility of a direct causal effect of one variable on another, carefully considering the direction of arrows. Excluding an arrow indicates an assumption of no direct causal influence."* | No | No |
| Grace & Irvine (2020) [3] | Focus on data-analysis | *“In this demonstration, we first considered a naïve causal diagram that considered all possible links. We used our naïve causal diagram and assembled expert knowledge to arrive at an informed diagram.”* | Consultation of experts is recommended, but no information on how experts are consulted. | No |
| Gaskell & Sleigh (2020) [4] | Focus on study design and data-analysis | *"How to Build a Directed Acyclic Graph in Seven Steps. 1. Define the primary causal relationship of interest and begin the causal diagram with the exposure and the outcome. 2. Insert important mediators; there may be more than one causal path between the exposure and the outcome. 3. Consider important causes of the exposure and important causes of the outcome (both measured and unmeasured). 4. Consider whether any two variables already on the directed acyclic graph share a common influence; if so this variable should be included. 5. Review variables in a pairwise manner—should any arrows be added? Absent arrows represent strong assumptions. 6. Ensure that any selection procedures are adequately captured. 7. Ensure that there are no feedback loops present; it may be necessary to include a variable at different time points (e.g., baseline, t1, t2) to maintain causality."* | No | No |
| Ferguson et al. (2020) [5] | Focus on data-analysis | *“It involves three key stages: (i) the conclusions of each study are ‘mapped’ into a DAG; (ii) the causal structures in these DAGs are systematically assessed using several causal inference principles and are corrected accordingly; (iii) the resulting DAGs are then synthesised into one or more ‘integrated DAGs’.”* | No | Yes |
| Suzuki et al. (2020) [6] | Focus on study design and data-analysis | *“(…) an arrow from P to Q is drawn when we suspect there is a direct causal effect (i.e., an effect not mediated through any other variables in the DAG) for at least one individual in the population, or when we are unwilling to assume*  *such individual causal effects do not exist. (…) To summarize, the presence or*  *absence of arrows in DAGs corresponds to the presence or absence of individual causal effect in the population.”; "Accordingly, when drawing DAGs, each researcher should carefully think about whether it even makes sense to include non-manipulable variables at all with any arrows into or out of them."; "Generally, drawing DAGs for the exposed or unexposed group should be avoided."* | No | No |
| Tennant et al. (2021) [7] | Focus on data-analysis | *"The DAG for a specific focal relationship should include all plausible confounding variables (i.e., that may plausibly cause both the exposure and the outcome), regardless of whether direct measurements are available or possible. Explicitly depicting unobserved variables helps to highlight potential sources of unobserved confounding."; "Variables should be visually arranged so that all constituent arcs flow in the same direction."; "Arcs should generally be assumed to exist between any two variables"; "Omitted arcs should therefore be carefully considered and ideally justified with theory and/or evidence."* | No | No |
| Watkins (2021) [8, 9] | Focus on study design and data-analysis | *“Specify/define the exposure (variable of interest) and the outcome as precisely as possible, including when their values have been or will be determined.”; “Specify/define all other variables for which data is available or is expected to be”; “For each variable, decide when the event occurred for each person that determined the value of that variable”; “Using the diagramming software of choice (or pen/pencil and paper), create the exposure and outcome variables in the diagram”; “Add all other variables and position them in the diagram so that those with data determined or recorded earlier in time are to the left of those determined later - Where they are positioned in relation to the exposure and outcome helps determine if they are potential confounders, mediators or colliders”; “Draw an arrow between any variables thought likely to be causally associated; indicating the direction of the causal relationship with the direction favouring the stronger causal effect if the variables affect each other over time but it is not clear which variable was determined earlier in the data”; “Do not draw an arrow between two variables if available knowledge and the plausibility of potential mechanisms suggests it is unlikely one may cause a meaningful change in the other. This also means that our research conclusions rest, in part, on our assumption that no causal relationship exists between them.”; “The causes of any one variable currently in the diagram may be included as additional (unmeasured) variables, but suspected causes of two or more variables should be included. This includes suspected unknown common causes of two or more variables, in which case a symbol such as U might serve as a label”* | No | Recommendation to check other articles that used a DAG for a similar research question |
| Laubach et al. (2021) [10] | Focus on data-analysis | *"The first step to drawing a DAG is to identify the X (cause) and Y (outcome) of interest."; "After defining the relationship of interest, the next step is to pinpoint sources of bias. One source of bias is confounders, or shared common causes of X and Y, denoted in a DAG as C with two arrows pointing at X and Y. Confounders are identified via prior knowledge in conjunction with evidence of a statistical association of C with X and Y in the study sample, and should typically be accounted for in order to obtain an unbiased causal effect of X on Y."; "Precision covariates are associated with X only or Y only, denoted as B pointing towards X or Y . Unlike confounders, there are no backdoor paths linking X to Y through B. Such variables account for technical variability and controlling for them in an analysis can improve model efficiency."* | No | No |
| Digitale et al. (2022) [11] | Focus on study design and data-analysis | *"To create a DAG one must specify: 1) the causal question of interest, thus necessitating inclusion of exposure or treatment (which we call E) and outcome of interest (D); 2) variables that might influence both E (or a mediator of interest) and D; 3) discrepancies between the ideal measures of the variables and measurements actually available to researchers; 4) selection factors that influence which patients are represented in the study population; and 5) potential causal relationships among these variables (depicted as arrows connecting variables). Even if a variable was not measured in the available data (or cannot be measured in most practical settings), it should nonetheless be represented in the DAG. Because the list of potential unmeasured variables can be long, a common convention visually simplifies by representing all unmeasured variables with the same causal structure (i.e., the same arrows in and out) as a single node."* | No | No |
| Hernán [12] | Focus on data-analysis | *"The causal DAG will obviously include a node for treatment A and a node for outcome Y. It will also include an arrow from A to Y to represent the causal effect that we are interested in."; "Very important, we are interested in the total effect of A on Y. Therefore, we don't need to specify the mechanisms through which A may affect Y. That is, we don't add to the graph any mediators"; "The next step is to add the causes shared by the treatment, A, and the outcome, Y, because the DAG that does not include the common causes is not a causal DAG."; "Remember, we need to add the common causes of every pair of variables in the DAG."; "Once we have added C (selection node), we also need to add the arrows to C from past variables, and from C to future variables."; "Every time we add a new variable to the DAG, we commit to adding all causes shared by that variable and any other variable already in the DAG."; "Finally, we need to add nodes for mismeasured variables."; “As always, we need to add common causes of the new node and any other variables already in the DAG”; "When drawing causal DAGs, we don't need to include all variables. As we have discussed, we only need to include the treatment, the outcome, the selection nodes, in some case the mediators, sometimes the measurement error, and always the common causes of any pair of variables in the graph."* | No | No |

**Bibliography**

1. Sauer B, VanderWeele TJ: **Use of directed acyclic graphs.** In *Developing a protocol for observational comparative effectiveness research: a user's guide.* Agency for Healthcare Research and Quality (US); 2013

2. Tafti A, Shmueli G: **Beyond overall treatment effects: Leveraging covariates in randomized experiments guided by causal structure.** *Information Systems Research* 2020, **31:**1183-1199.

3. Grace JB, Irvine KM: **Scientist’s guide to developing explanatory statistical models using causal analysis principles.** *Ecology* 2020, **101:**e02962.

4. Gaskell AL, Sleigh JW: **An introduction to causal diagrams for anesthesiology research.** *Anesthesiology* 2020, **132:**951-967.

5. Ferguson KD, McCann M, Katikireddi SV, Thomson H, Green MJ, Smith DJ, Lewsey JD: **Evidence synthesis for constructing directed acyclic graphs (ESC-DAGs): a novel and systematic method for building directed acyclic graphs.** *International journal of epidemiology* 2020, **49:**322-329.

6. Suzuki E, Shinozaki T, Yamamoto E: **Causal diagrams: pitfalls and tips.** *Journal of epidemiology* 2020**:**JE20190192.

7. Tennant PW, Murray EJ, Arnold KF, Berrie L, Fox MP, Gadd SC, Harrison WJ, Keeble C, Ranker LR, Textor J: **Use of directed acyclic graphs (DAGs) to identify confounders in applied health research: review and recommendations.** *International journal of epidemiology* 2021, **50:**620-632.

8. Watkins T: **1070 An online searchable database of example causal diagrams to make them easier to construct.** *International Journal of Epidemiology* 2021, **50:**dyab168. 702.

9. **Causal diagrams. Learn by example** [<https://causaldiagrams.org/>]

10. Laubach ZM, Murray EJ, Hoke KL, Safran RJ, Perng W: **A biologist's guide to model selection and causal inference.** *Proceedings of the Royal Society B* 2021, **288:**20202815.

11. Digitale JC, Martin JN, Glymour MM: **Tutorial on directed acyclic graphs.** *Journal of Clinical Epidemiology* 2022, **142:**264-267.

12. **Causal Diagrams: Draw Your Assumptions Before Your Conclusions** [<https://www.edx.org/course/causal-diagrams-draw-your-assumptions-before-your>]
